# Mapping Genetic Variants Underlying Differences in the Central Nitrogen Metabolism in Fermenter Yeasts

**DOI:** 10.1371/journal.pone.0086533

**Published:** 2014-01-21

**Authors:** Matías Jara, Francisco A. Cubillos, Verónica García, Francisco Salinas, Omayra Aguilera, Gianni Liti, Claudio Martínez

**Affiliations:** 1 Departamento de Ciencia y Tecnología de los Alimentos, Universidad de Santiago de Chile (USACH), Santiago, Chile; 2 Centro de Estudios en Ciencia y Tecnología de Alimentos (CECTA), Universidad de Santiago de Chile (USACH), Santiago, Chile; 3 Institute for Research on Cancer and Aging of Nice (IRCAN), Université de Nice Sophia Antipolis, Nice, France; University of Iceland, Iceland

## Abstract

Different populations within a species represent a rich reservoir of allelic variants, corresponding to an evolutionary signature of withstood environmental constraints. *Saccharomyces cerevisiae* strains are widely utilised in the fermentation of different kinds of alcoholic beverages, such as, wine and sake, each of them derived from must with distinct nutrient composition. Importantly, adequate nitrogen levels in the medium are essential for the fermentation process, however, a comprehensive understanding of the genetic variants determining variation in nitrogen consumption is lacking. Here, we assessed the genetic factors underlying variation in nitrogen consumption in a segregating population derived from a cross between two main fermenter yeasts, a Wine/European and a Sake isolate. By linkage analysis we identified 18 main effect QTLs for ammonium and amino acids sources. Interestingly, majority of QTLs were involved in more than a single trait, grouped based on amino acid structure and indicating high levels of pleiotropy across nitrogen sources, in agreement with the observed patterns of phenotypic co-variation. Accordingly, we performed reciprocal hemizygosity analysis validating an effect for three genes, *GLT1*, *ASI1* and *AGP1*. Furthermore, we detected a widespread pleiotropic effect on these genes, with *AGP1* affecting seven amino acids and nine in the case of *GLT1* and *ASI1*. Based on sequence and comparative analysis, candidate causative mutations within these genes were also predicted. Altogether, the identification of these variants demonstrate how Sake and Wine/European genetic backgrounds differentially consume nitrogen sources, in part explaining independently evolved preferences for nitrogen assimilation and representing a niche of genetic diversity for the implementation of practical approaches towards more efficient strains for nitrogen metabolism.

## Introduction

Yeast strains isolated from various geographic origins have long been recognised as the main microorganisms underlying a number of fermentation processes [1]. In this line, *Saccharomyces cerevisiae* isolates have been widely utilised in the industry for the alcoholic fermentation of different kinds of beverages, representing a rich reservoir of genetic variants selected upon their fermentation capacity within these environments [2]. The Saccharomyces Genome Resequencing Project (SGRP) [2] revealed a vast genetic diversity among natural and industrial isolates, defining five clean populations as well as strains of mixed ancestry. Two of these lineages contain strains from separated locations, but common usage in industrial fermentations (wine and sake), demonstrating the importance of the baker’s yeast in historical human activities [1], [3].

Wine fermentation corresponds to a complex biochemical and microbiologic process where many organisms are involved [4]–[5]. Nonetheless, *S. cerevisiae* is the principal species responsible for the alcoholic fermentation, producing and tolerating high ethanol levels [4]–[6]. During this process, *S. cerevisiae* is exposed to severe osmotic stresses with high sugar concentrations and low availability of nitrogen respect to carbon. To overcome these limitations, yeasts have developed different mechanisms, such as, specific permeases to efficiently metabolise some sugar compounds [7]–[8], ethanol resistance strategies and a complex regulation mechanism for nitrogen utilisation [9]–[10]. Yeasts can utilise a wide diversity of nitrogen compounds [11]. The amount of nitrogen available in the grape must vary across samples, depending on climate conditions and the grape fertilisation system, among others [12]. Additionally, adequate nitrogen levels in the medium are essential for a good fermentation, since low levels can prompt slow fermentations or even stop the process, causing important economic losses [13]. To avoid this problem, grape must is sometimes supplemented with ammonium salts, however, excesses of inorganic nitrogen can induce the production of toxic ethyl carbamate and hence alter yeasts’ amino acid preferences and wine aromatic properties [14]. These facts highlight the need for superior strains with efficient yields on nitrogen consumption to reduce practices that could alter the final fermentation product.

The grape must predominantly contains two nitrogen sources, amino acids and ammonium, which yeasts consume through active transport and specific permeases [15]. Subsequently, the nitrogen enters the central nitrogen metabolism pathway as ammonium. This nitrogen source can be directly consumed or produced from amino acids through transamination, where a glutamate dehydrogenase (*GDH1* & *GDH3*) incorporates the ammonium to α-ketoglutarate or to glutamate through the action of glutamine synthase (*GLT1*) and generating glutamine [16]–[17]. In addition, nitrogen sources are consumed based on their capacity of being assimilated in the central nitrogen metabolism [11], that is regulated by the nitrogen catabolic repression system [18]. Among the main players involved in the central nitrogen metabolism, the action of *GAP1* and the *MEP* gene family coding for ammonium permeases has been demonstrated to be an important adaptation event for the fermentation conditions [10], [19]. Similarly, expression studies during must fermentation validated the role of *GAP1* upon nitrogen transport and reported expression changes for *GDH2*, *GDH3* and *PUT1* involved in metabolism and *GCN4* and *MET30* in regulation [20]. Altogether, these processes explain the complexity of nitrogen consumption and its robust regulation, representing a valid tool for the study of the allelic variants and the phenotypic differences between strains for nitrogen assimilation [17], [21].

The study of the allelic variants in diverse isolates underlying nitrogen consumption during the alcoholic fermentation process allows gaining insight into the adaptation mechanisms developed to withstand the fermentation conditions where nitrogen is low [13]. Moreover, the identification of alleles conferring adaptive advantages promises new applications in the biotechnology industry, where these genetic variants can be used as trait markers [22]. Several studies on proteome and genome sequence analysis have described unique genetic polymorphisms in wine isolates, likely resulting from the adaptation to the fermentative process [23]–[25]. Recently, the mapping of quantitative trait loci (QTLs) on oenological traits has proven fruitful in a series of studies, providing evidence of the allelic variants underlying the adaptation of wine yeasts for traits such as, malic acid and succinic acid production, ethanol accumulation, nitrogen metabolism and residual sugar, among others [13], [26]–[28]. Similarly, genomic regions underlying wine aroma diversity were recently described in a S288c×59a cross, the latest an haploid strain derived from the commercial wine isolate EC1118, identifying principally the para-aminobenzoate (PABA) synthase and the eQTL hotspot *ABZ1* underlying the production of numerous aromatic compounds [29]–[30]. On the other hand, although many other strains have been long associated with fermentation processes of varied sources, reports on their genetics and potentially independently evolved mechanisms to tolerate the fermentation processes are scarce [3]. For example, only few studies have used sake strains to follow their fermentation capacity, majority focused on their superior ethanol tolerance [28], [31].

In the present study, we used 96 segregants from a cross between a Wine European isolate (DBVPG6765) and a Sake strain (Y12) [26], [32] to investigate the molecular bases of the genetic variation underlying nitrogen metabolism traits during fermentation. The population of segregants was phenotyped after six days of fermentation in synthetic must for amino acids and ammonium consumption. Differences among segregants were primarily based for preferences on amino acid structure. Linkage mapping revealed the presence of many QTLs, majority of which corresponded to pleiotropic genes involved in the consumption of several amino acids. Moreover, we validated three genes by reciprocal hemizygosity and demonstrate how, Sake and Wine/European genetic backgrounds, have independently evolved distinctive nitrogen sources preferences, providing new evidence on the means by which genetic variation shapes fermentation processes associated to human activities.

## Results

### High Phenotypic Variation for Total Nitrogen Consumption

In order to characterise nitrogen metabolism traits in fermenter yeasts, initially DVBPG6765 (WE) and Y12 (SA), two strains belonging to clean lineages of *S. cerevisiae* associated to human activities [2], were grown in synthetic wine must (MS300). We divided the total amount of nitrogen into 15 different traits corresponding to ammonium and 14 amino acids, because these are known to be used as nitrogen sources at this stage [33]. We estimated total consumption levels after six days of fermentation (**Table 1**). This time point corresponds to an intermediate fermentation stage where most changes for nitrogen consumption have occurred [33]–[34]. We observed that nitrogen sources were differentially consumed depending on the strain, with HPLC profiles showing significant differences for thirteen amino acids and ammonium (FDR <5%, **Table 1**). For example, over 90% of initial MS300 aspartic acid, glutamic acid, methionine and lysine were consumed in the SA strain, representing highly preferred sources. Likewise, WE consumed high levels of aspartic acid and glutamic acid, nevertheless and contrasting SA, leucine and phenylalanine were prominently consumed in the WE strain. For those sources with a significant difference between strains, the WE strain showed a preference for ammonium utilisation as nitrogen source, representing 52.3% of the total nitrogen consumed, in contrast to SA for which ammonium only represents 35.2% of the total nitrogen. On the other hand, amino acids and particularly glutamine were the source prominently used by SA (64.8% and 18.7%, respectively), representing the main source of nitrogen for this strain. These differences demonstrate that the two strains have individual nitrogen preferences and a wide phenotypic diversity in regard to nitrogen consumption sources.

**Table 1:** Consumption levels in parental strains and F1 hybrids of each nitrogen source.

Nitrogen Source	SA R1	SA R2	SA_Av	% Initial	% YAN	WE R1	WE R2	WE	% Initial	%YAN	HybridR1	HybridR2	Hybrid	% Initial	% YAN	ANOVAp-valuesSA vs WE	q-values
Ammonium	169.7	158.9	164.3	35.7	35.2	316.5	311.5	314.0	68.3	52.3	301.6	286.8	294.2	64.0	48.5	0.002	0.0022*
Arginine	100.8	94.0	97.4	19.3	5.3	61.3	48.5	54.9	10.9	2.3	98.3	93.6	95.9	19.1	4.0	0.028	0.0144*
Aspartic acid	44.4	44.5	44.5	99.9	3.8	44.0	44.0	44.0	98.8	2.9	44.4	44.4	44.4	99.7	2.9	0.018	0.0100*
Phenylalanine	26.0	26.0	26.0	68.6	1.8	36.0	36.1	36.1	95.2	1.9	35.9	35.9	35.9	94.7	1.9	0.000	0*
Glutamate	117.4	117.9	117.7	97.8	9.2	115.0	115.2	115.1	95.6	7.0	116.0	116.0	116.0	96.3	7.0	0.010	0.0061*
Glutamine	239.2	236.6	237.9	47.1	18.7	223.5	216.0	219.7	43.5	13.4	242.7	238.4	240.5	47.6	14.5	0.044	0.0214*
Histidine	21.8	22.2	22.0	44.0	1.2	37.9	40.1	39.0	78.0	1.7	38.0	37.6	37.8	75.6	1.6	0.004	0.0042*
Isoleucine	22.1	22.4	22.2	68.0	1.9	28.1	28.9	28.5	87.1	1.9	29.1	29.4	29.2	89.4	2.0	0.004	0.0042*
Leucine	40.2	41.1	40.7	84.1	3.6	46.5	46.7	46.6	96.2	3.2	47.0	47.3	47.2	97.4	3.2	0.006	0.0045*
Lysine	23.6	23.5	23.5	98.1	1.5	18.7	18.2	18.5	76.9	0.9	21.4	21.4	21.4	89.2	1.0	0.002	0.0022*
Methionine	28.2	28.2	28.2	89.9	2.2	27.6	27.6	27.6	88.0	1.6	27.6	27.4	27.5	87.5	1.6	0.002	0.0022*
Serine	27.4	26.6	27.0	34.4	2.9	19.3	18.2	18.8	23.9	1.6	26.4	26.3	26.4	33.6	2.2	0.007	0.0046*
Tyrosine	2.7	2.0	2.4	12.9	0.1	7.1	7.3	7.2	39.5	0.4	5.2	5.2	5.2	28.4	0.3	0.006	0.0045*
Threonine	34.3	34.2	34.2	45.1	3.3	27.2	26.9	27.1	35.7	2.0	33.5	34.3	33.9	44.7	2.5	0.000	0.0016*
Tryptophan	167.4	157.6	162.5	90.6	9.3	159.0	152.4	155.7	86.8	6.9	159.3	148.7	154.0	85.9	6.8	0.368	0.1665

Amount of nitrogen source consumed (mg/L), the relative percentage respect to the initial amount, its relative contribution to the total amount of nitrogen consumed and ANOVA statistical analysis in parental and F1 hybrid strains are shown. R1 and R2 represent each individual replicate, % Initial refers to the amount consumed respect to the initial amount provided and % YAN refers to the amount consumed respect to the total nitrogen provided. Av = average.

**Figure 1:**
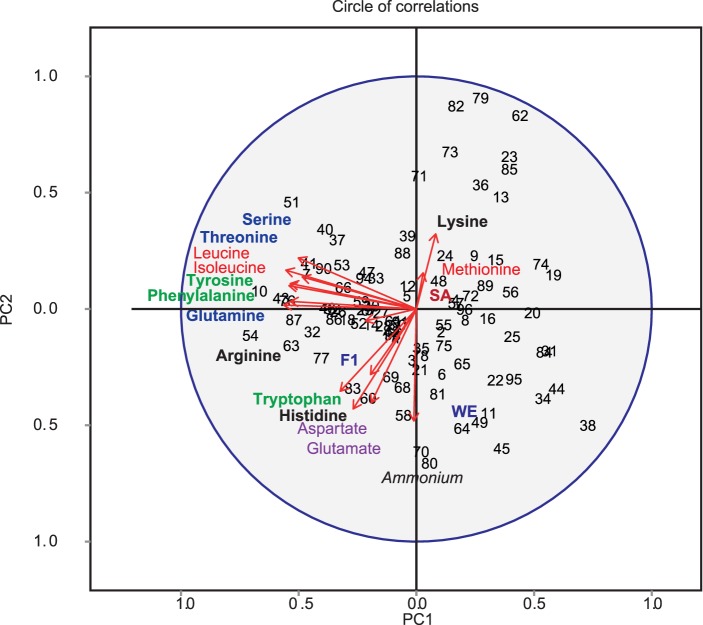
A Principal Component Analysis of ammonium and amino acid consumption variation across segregants. Repartition of the 14 amino acids and ammonium are shown on the PC1 and PC2 axis. PC1 explains 40% of the variation, while PC2 17%. Amino acids, Red: Non-polar, Blue: Polar uncharged side chain, Black: Polar positively charged side chain, Purple: Polar negatively charged side chain, Green: Aromatic.

Using a segregating population derived from the cross of these two parental strains [32], we estimated levels for the previously mentioned 15 traits in synthetic wine must for 96 individuals. Fermentations were carried out in duplicates, with two completely independent biological replicates for each segregant [26], [30] (**Table S1**). All phenotypes showed a continuous distribution, suggesting a polygenic contribution (**Figure S1**). An identified genetic association for a given phenotype could evidence associations for other correlated phenotypes and reflect shared genetic covariances [35]. To evaluate the correlation between phenotypes, we performed a global principal component analysis (**Figure 1A**). The first factorial plane defined by the PC1 and PC2 components, accounted for 40% and 17% of the observed variation respectively. The correlation circle generated shows that amino acids mostly grouped based on their structure (negatively or positively charged, polarity and aromatic side chain), with PC2 particularly explaining ammonium preference as nitrogen source. Altogether, these two variables explain 57% of the overall variation.

Unlike the parental strains, we found segregants able to efficiently consume several nitrogen sources, better than their parents (**Figure S1**). In this regard, we found high transgression levels in all traits (percentage of segregants exceeding the phenotypic range of their parents by at least 2 SD), ranging from 19 to 78 segregants for histidine and methionine, respectively. The presence of transgressive segregants could reflect that alleles with opposite effects or other genetic interactions, such as epistasis or overdominance, are present within the genetic configuration of the parental strains. The phenotypic diversity described together with the high number of transgressive segregants proves that this recombinant population is well suited for linkage analysis to determine genetic variation linked to nitrogen consumption under fermentative conditions.

### High Levels of Pleiotropy for Nitrogen Consumption in WE × SA Segregants

**Figure 2:**
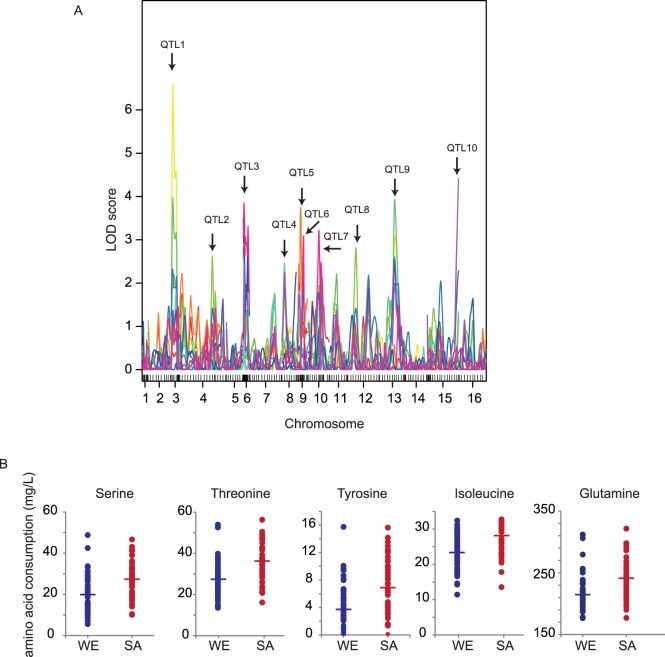
Linkage analysis in WE x SA cross. **A.** LOD plot from linkage analysis using a nonparametric model for all traits with at least a single QTL. QTLs for the corresponding phenotype are: QTL1, Serine and Threonine; QTL2, Glutamic Acid; QTL3, Lysine and Tryptophane; QTL4, Phenylalanine and Tyrosine; QTL5, Aspartate and Glutamate; QTL6, Ammonium and Lysine; QTL7, Lysine; QTL8, Histidine; QTL9, Isoleucine, Serine, Tyrosine, Glutamine and Threonine; QTL10, Phenylalanine and Tyrosine. **B.** Amino Acid consumption levels in segregants carrying either WE or SA alleles for QTL XIII.527 underlying Isoleucine, Serine, Tyrosine, Glutamine and Threonine variation.

To investigate the genetic architecture underlying nitrogen consumption variation in isolates of *S. cerevisiae*, we performed linkage analysis of the trait variations described in the preceding section. We applied single interval mapping (IM) utilising the genotype information from our previous studies [26], [32]. We discovered several regions involved in the natural diversity for the utilisation of amino acids and ammonium as nitrogen sources. We identified 18 QTLs for 12 out of 15 phenotypes analysed (FDR <5%, **Table S2**, **Figure 2A**). Of these, seven QTLs controlled more than two traits, suggesting that pleiotropic effects are common. For example, we found a common region (QTL1) for two traits: serine and threonine, two polar amino acids with a hydroxyl group, and representing the strongest QTL of the study explaining 29.1% and 16.5% of the trait’s variation respectively (**Figure 2B**, **Table S2**). Furthermore, we found other six QTLs involved in at least two traits, indicating high pleiotropic levels in amino acid consumption.

Specific QTLs for amino acids with similar structure were also found. Phenylalanine and tyrosine are two amino acids with an aromatic and hydrophobic side chain exhibiting significant phenotypic correlation among segregants (Pearson = 0.781, *P*<0.001) and both controlled by QTL10. Yet, one additional QTL (XIII.527, QTL9) was mapped for tyrosine explaining 6.8% of the trait’s variation and suggesting different genetic control mechanisms. Similarly, glutamic acid and aspartic acid are two negatively charged amino acids and share a single QTL (IX.149, QTL5) explaining 18.5% and 16.7% of the trait’s variation respectively. Still, a specific QTL (IV.101, QTL2) for glutamic acid was found explaining 9.3% of the phenotype’s variation. Nevertheless, at a more lenient threshold (FDR = 15%), this region was also observed for aspartic acid explaining over 8.2% of the variation (data not shown). Though, differences were observed between parents for methionine and leucine, we were not able to identify QTLs for these amino acids, likely due to a complex polygenic segregation which could only be detected using a greater progeny size or alternative mapping methods [36].

In those nitrogen sources highly favoured in synthetic grape must by WE and SA parental strains (ammonium and glutamine, respectively), we only found one QTL (XIII.527, QTL9) for glutamine at a 5% FDR. In agreement with the tendency observed for that in parents, strains carrying the SA allele tended to consume greater levels of glutamine than those with the WE allele (**Figure S2A**). Although no QTLs were found for ammonium at 5% FDR, a slightly more lenient threshold (FDR = 7%) revealed the presence of QTL6 underlying 11.3% of the phenotype’s variation (**Table S2**). Segregants carrying the WE allele showed higher ammonium consumption levels compared to those bearing the SA allele (**Figure S2B**), in agreement with parental preferences. Nevertheless, the lack of QTLs at lower FDR suggests that many loci could underlie variations in glutamine and ammonium consumption between SA and WE strains.

A recombinant set of segregants allows detecting antagonistic alleles which can increase fitness even though they come from an unfit strain, or vice versa [37]. Although the SA strain has superior glutamic acid consumption levels, we found that segregants carrying the WE allele for QTL5 consumed greater levels of this amino acid. This antagonistic tendency was also found for another six QTLs (**Table S2**), demonstrating how pervasive this effect can be.

### *GLT1* Underlies Higher Aspartic and Glutamic Acid Consumption in the SA Strain

**Figure 3:**
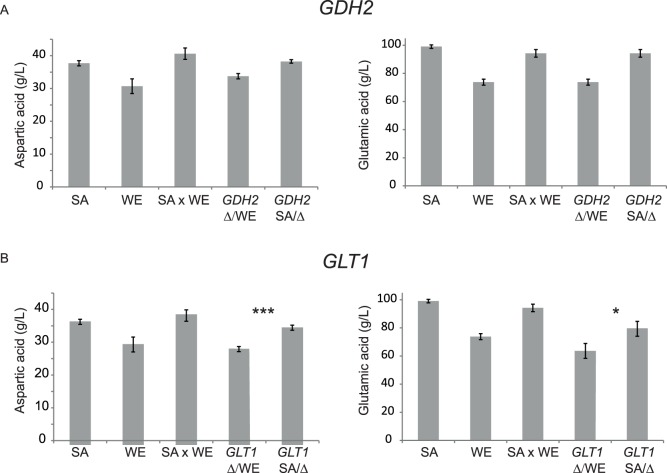
Reciprocal hemizygosity analysis on *GHD2* and *GLT1* underlying consumption variation for Aspartic and Glutamic acid. **A.** The hybrid hemizygote strains consumption levels (mg/L) for SA and WE *GDH2* reciprocal hemizygotes. **B.** Similarly, *GLT1* reciprocal hemizygosity assay. (*), (**) and (***) represents a significant statistical difference between the hemizygote strains for the same gene using ANOVA tests *P*<0.05, *P*<0.01 and *P*<0.001 respectively. Δ/WE denotes hemizygotes carrying the WE allele, while SA/Δdenotes hemizygotes carrying the SA allele.

In order to identify causative loci for traits of interest, we further studied in detail QTL2 and QTL5 underlying aspartic and glutamic acid consumption differences, because these two amino acids were highly preferred by both strains. For this, we examined polymorphisms’ data from the SGRP and YRC databases, gene function descriptions from SGD and performed a GO term search over a set list of nitrogen related genes. Initially, we filtered genes based on the presence of single nucleotide polymorphism within the ORF or regulatory regions between both strains and selected several genes adjacent to the QTLs. Next, we looked at those genes with function descriptions which could be most likely related to nitrogen metabolism. Based on this filtering, we narrowed down the gene list to two candidates in QTL2, *GDH2* and *GLT1*, while no adequate candidate genes were found for QTL5 under these criteria. *GDH2* is a NAD(+)-dependent glutamate dehydrogenase which degrades glutamate to ammonia, while *GLT1* a glutamate synthase that synthesizes glutamate from glutamine. For the validation of the candidate genes, we performed Reciprocal Hemizygosity Analysis (RHA) by independently deleting one allele at a time of the candidate gene in the original WE × SA hybrid. Each *GLT1* and *GDH2* reciprocal hemizygote was fermented during six days in quintuplicates to address the effects of the WE and SA alleles in aspartic and glutamic acid consumption. Our results on *GDH2* showed a non-significant allelic difference for the consumption of glutamic acid (*P = *0.03 ANOVA, FDR = 0.13,) and aspartic acid (*P* = 0.04 ANOVA, FDR = 0.13) (**Figure 3A**). RHA on *GLT1* showed a different outcome, with significant allelic differences at a 1% FDR for aspartic acid (*P*<0.0001, ANOVA) and glutamic acid (*P*<0.01, ANOVA) (**Figure 3B**), indicating that *GLT1* is the main causative gene for higher glutamic acid and aspartic acid consumption levels in the SA strain. Although *GLT1* is linked to QTL2, a region that mapped solely for glutamic acid, the RHA also showed differences in consumption levels for other seven amino acids (serine, glutamine, threonine, tryptophan, leucine, lysine and isoleucine, **Table S3**), suggesting a pleiotropic effect.

Likewise, we attempted to narrow down the number of genes on QTL3 underlying tryptophan and lysine consumption. Within this QTL we found the candidate gene *AGP3*, a low-affinity amino acid permease, harboring functional polymorphisms in the SA background and suggesting amino acids uptake differences. Nevertheless, we could not obtain significant differences between reciprocal hemizygotes for the consumption of any amino acid (**Table S3**), indicating that the right candidate gene for this region was not identified.

### *AGP1* and *ASI1* are Pleiotropic Genes Involved in Several Amino Acids Consumption Variation

**Figure 4:**
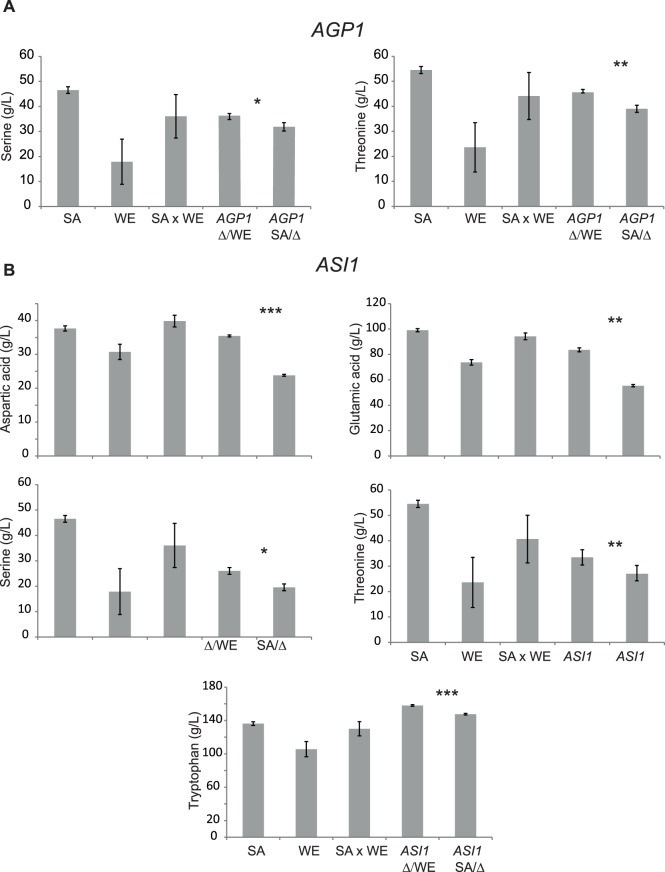
Reciprocal hemizygosity analysis on *AGP1* and *ASI1*. **A.** The hybrid hemizygote strains serine and threonine consumption levels (mg/L) for SA and WE *AGP1* reciprocal hemizygotes. **B.** Aspartic acid, glutamic acid, serine, threonine and tryptophane consumption levels (mg/L) for *ASI1* reciprocal hemizygotes. (*), (**) and (***) represents a significant statistical difference between the hemizygote strains for the same gene using an ANOVA test with *P*<0.05, *P*<0.01 and *P*<0.001 respectively. Δ/WE denotes hemizygotes carrying the WE allele, while SA/Δ denotes hemizygotes carrying the SA allele.

The majority of QTLs found on this study were mapped for polar amino acids. Therefore, we next analysed QTLs underlying differences in serine, threonine and glutamine consumption levels. Initially, we focused on QTL1, the largest effect QTL of the study mapped for serine and threonine uptake. To validate the effect of this region, we applied a similar gene filtering strategy as in the previous section and identified the promising candidate *AGP1* for RHA validation, an amino acid permease involved in the uptake of several amino acids. The fermentation profile of the WE-*agp*1/SA-*AGP1* reciprocal hemizygote showed lower levels of serine (*q* <0.01, ANOVA), threonine (*q* <0.008, ANOVA) and glutamine (*q* <0.0001, ANOVA) consumption levels respect to the WE-*AGP1*/SA-*agp1*Δ hemizygote (**Figure 4A**), in agreement with the tendency observed in the recombinant population. Moreover, other amino acids also displayed a similar pattern, with significant differences for phenylalanine, aspartic acid and arginine (**Table S3**), validating the presence of pleiotropic factors involved in nitrogen metabolism.

Next, we focused on QTL9, the most pleiotropic region of the study with significant linkage for glutamine, isoleucine, serine, threonine and tryptophan (**Figure 2**). Here, we identified two candidate genes, *ASI1* a putative integral membrane E3 ubiquitin ligase involved in the SPS-sensing pathway to respond to extracellular amino acids and *GAT2*, a gene encoding for a protein containing GATA family zinc finger motifs and induced by leucine. RHA showed that polymorphisms in *GAT2* had no significant effect on any amino acid (**Table S3**). The construction of reciprocal hemizygotes for *ASI1* showed differences for several amino acids, including aspartic acid (*q* <0.0005, ANOVA), glutamic acid (*q* <0.002, ANOVA), serine (*q* <0.01, ANOVA), threonine (*q* <0.009, ANOVA), and tryptophane (*q* <0.0005, ANOVA) (**Figure 4B**). These results, where WE-*ASI1*/SA-*asi1* consumed greater levels of the above mentioned amino acids respect to WE-*asi1*/SA- *ASI1* hemizygotes, were in opposite direction from those obtained in the recombinant population and suggest that other loci within the region could also underlie amino acid consumption.

### Polymorphisms in the Validated Genes *GLT1*, *AGP1* and *ASI1*

To identify the polymorphisms that could underlie the allelic effects observed in the reciprocal hemizygotes strains, we further analysed the changes within the regulatory and coding regions of *GLT1*, *AGP1* and *ASI1* by examining the SGRP database [38] and SIFT analysis results [38]. A SIFT analysis allows to predict if an amino acid substitution could significantly affect a protein domain based on sequence homology analysis and the physical properties of the amino acid changes.

Sequence comparison analysis on the *GLT1* sequence revealed four non-synonymous polymorphisms in the WE background (G65E, D321N, K679R and G1988D) affecting several domains of the protein. A SIFT analysis identified that G1988D could be deleterious, affecting the FAD/NAD(P)-binding domain. This amino acid change could likely affect the protein’s function and explain the lower ability of the WE allelic variant to metabolise glutamic and aspartic acid.

We detected in the *AGP1* gene five non-synonymous polymorphisms (L7P, G24E, Q92R, A530S and D597N), all of them in the SA strain, except for L7P. Domain prediction analysis allowed us to find a sole change, A530S, affecting the amino acid permease domain, while the SIFT analysis did not find deleterious candidates. Nevertheless, the other four polymorphisms represent changes of unrelated amino acids and likely G24E, Q92R and D597N could affect the protein structure and therefore the ability of the SA variant to metabolise polar amino acids. We identified two non-synonymous changes between *ASI1^WE^* and *ASI1^SA^* (K550E & T557I). Since none of these changes falls within a particular domain and the SIFT analysis did not predict deleterious modifications, the sole charge modification from lysine to glutamic acid (K550E) in *ASI1^WE^* represents the strongest candidate underlying the observed phenotypic differences (**Figure 4B**). Finally, in order to determine the distribution of alleles across other yeast isolates, we generated a phylogenetic tree obtained from a concatenated analysis of *GLT1*, *AGP1* and *ASI1* (**Figure S3**). The sequences of the WE and SA strains fall within distinct clusters, in agreement with their niche specificity and previously described phylogenetic topologies [2]. Interestingly, besides those strains formerly shown to belong to these clusters, no other strains (except for Y55 clustered with the WE strains) were observed to group with either WE or SA strains, indicating unique allelic configurations for the latter mentioned clusters.

## Discussion

We have studied baker’s yeast nitrogen consumption profiles in a previously generated cross between a Wine/European (WE, DBVPG6765) and a Sake (SA, Y12) isolate [26], [32]. Although these two strains originated from different geographical regions, both of them have been associated to natural fermentation processes in close proximity to human activities [2]. While the WE isolate clustered well with strains obtained from grape juice or European regions, the SA strain gathered with those isolated in Japan from rice fermentations. Bearing in mind the heterogeneous origins of these strains, we would expect differences in their ability to ferment diverse substrates since these isolates likely underwent different selective pressures. Certainly, dissection of nitrogen consumption in amino acids and ammonium after six days of fermentation in synthetic wine must allowed us to identify individual preferences for each strain (**Table 1**). After six days of fermentation cumulative CO_2_ weight lost data for this cross indicates that some individuals may be at different physiological states (*Araos*, unpublished data). Although we are aware that this may be a concern for our analysis, we expect that changes for nitrogen consumption have predominantly ceased and no secretion of other nitrogen compounds is noticeable yet, representing an affinity related effect [34].

While both, the SA and WE strains, consumed over 90% of negatively charged amino acids, the rice fermentation isolate utilised the majority of methionine and lysine, whereas WE better utilised leucine and phenylalanine, reflecting majority of variation in sources generally present in low concentrations. Moreover, for those nitrogen sources highly supplemented in the medium, ammonium represents the preferred source by WE, in contrast to SA, where amino acids, particularly glutamine, were the prominent utilised source. Our results agree with recent findings on which high-resolution phenotype analysis on nitrogen source preference and utilization revealed a wide diversity among wine strains [13]. In our study, the SA preference on amino acids and WE on ammonium demonstrate that the two strains may have developed an independent adaptive response to alcoholic fermentation by opting for different nitrogen sources likely determined by their substrate availability. Indeed, it has been reported that rice wine is rich in nitrogen derived from amino acids (rather than ammonium), which may explain the observed 65% SA consumption preference of amino acids in lieu of ammonium of the total assimilated nitrogen (**Table 1**) [39]. Moreover, niche variations can have a large impact on trait divergence [21], [40]–[42], supporting our findings of individual nitrogen consumption profiles.

The identification of alleles underlying adaptive advantages and explaining widespread population variation differences represents a main challenge in current biology [43]. To determine the molecular bases behind nitrogen utilisation in synthetic must (MS300), we phenotyped 96 segregants derived from the WE × SA hybrid for 15 nitrogen sources. The PCA analysis demonstrated that consumption is highly dependent on the amino acid structure, explaining over 57% of the observed variation (**Figure 1A**). Following our PCA and trait correlation analyses, differences between strains appeared relevant to perform QTL mapping. Linkage analysis showed that out of 18 QTLs identified, seven underlie more than one trait, revealing a total number of 10 QTLs across traits. For example, our data shows significant quantitative associations of QTL4 to phenylalanine and tyrosine, being in all cases the WE allele responsible for greater levels of nitrogen consumption (**Figure 2A**). Pleiotropy of this sort has been long observed in mutant analyses, identifying thousands of genes affecting multiple traits [44]–[45]. In this line, it has been proposed that pleiotropic genes could have detrimental disadvantages for an individual by limiting its adaptation capacity, unless correlated to beneficial traits, also denominated as antagonistic pleiotropy [44], [46]–[47]. In our study, majority of common QTLs among traits could be group based on amino acid structure, in agreement with the observed patterns of phenotypic co-variation and indicating co-evolution of traits [48]. Part of the detected pleiotropy in our study could be explained by the fact that amino acids with similar structure converged into the same pathway under the control of common factors [49], along with functional relationships to co-ordinately regulate nitrogen assimilation [50]. Another possible explanation for the observed pleiotropy is that most amino acid permeases can transport more than one type of amino acid. For example, Bap2p and Bap3p permeases mainly transport branched amino acids, Agp1p branched polar, branched non-polar amino acids and even some aromatic amino acids [17], [49].

To identify the allelic variants underlying nitrogen assimilation differences between SA and WE strains, we looked for candidate loci within QTLs. To this end, we tried to narrow down the number of genes by utilising a filtering strategy combining the SGD gene description and polymorphisms data from SGRP and YRC databases [2], [38], [51]. Using this approach, we identified candidate genes, *AGP1* for QTL1, *GDH2* and *GLT1* for QTL2, *AGP3* for QTL3 and *ASI1* and *GAT2* for QTL9. Subsequently, we performed reciprocal hemizygosity analysis and validated an effect for *GLT1*, *AGP1* and *ASI1*. Furthermore, the reciprocal hemizygosity analyses allowed us to validate the pleiotropic effects of this set of genes, with seven amino acids affected by *AGP1* and nine for *GLT1* and *ASI1*, demonstrating their widespread effect. Interestingly, the alleles we identified were not involved among the previously identified QTLs on any wine or sake-related study [13], [26]–[29], [31]. Yet, complex genetic architecture on oenological traits in the form of many genes contributing to a single phenotype was reported in these studies. In our study, the *GLT1^SA^* variant increases aspartic and glutamic acid consumption (**Figure 3B**). We found that the G1988D variant on *GLT1^WE^* (a NAD(+)-dependent glutamate synthase, that synthesizes glutamic acid from glutamine and α-ketoglutarate) could alter the enzyme’s function by decreasing the assimilation of all those amino acids whose ammonium ion is assimilated through glutamic acid transamination to generate glutamine. In fact, SA-*glt1*/WE-*GLT1* reciprocal hemizygote consumes lower levels of threonine, aspartic acid, glutamic acid, tryptophane and isoleucine (**Figure 3B**). On the other hand, we only found a significant effect for *GDH2* at a lenient 13% FDR threshold for aspartic acid and glutamic acid, which does not allow us to make further conclusions. *GDH2* codes for a NAD(+)-dependent glutamate dehydrogenase involved in the central nitrogen metabolism, which degrades glutamic acid to ammonia and α-ketoglutarate [18].

The *AGP1^SA^* and *ASI1^SA^* alleles reduce the assimilation of polar amino acids, such as, serine, threonine and glutamine, the negatively charged aspartic acid and the aromatic amino acid phenylalanine (**Figure 4**). *AGP1^SA^*, a broad-specificity amino acid permease [49], [52], carries an A530S polymorphism affecting the permease domain and therefore likely modifying several amino acids uptake. Indeed, the reciprocal hemizygote SA-*AGP1*/WE-*agp1*Δ make use of a lower amount of glutamine and arginine, two of the main *AGP1* substrates, as well as others amino acids also transported by *AGP1*, such as, serine, threonine and phenylalanine [49]. The *ASI1* gene encodes an inner nuclear membrane protein capable of recruiting unprocessed N-terminal Stp1p/Stp2p transcription factors, hindering their binding on promoter regions and thus impeding gene expression on transporters involved in amino acid intake [49], [53]. Moreover, this system also regulates gene expression for other permeases, such as, *AGP1*, *BAP2*, *BAP3*, *GNP1* and *TAT1*, selectively sensing through the Sys system extracellular free amino acids [54]. We identified that *ASI1* ^WE^ contains a K550E polymorphism that could affect *ASI1* function by impeding its regulation upon Stp1p/Stp2p transcription factors. In this way, *ASI1* would induce permeases gene expression, augmenting amino acid consumption levels as observed in the SA-*asi1*/WE-*ASI1* reciprocal hemizygote for nine amino acids. Interestingly, the greater uptake of these amino acids by *ASI1^WE^* allele is in contrast to the parental phenotype, representing an antagonistic locus.

**Figure 5:**
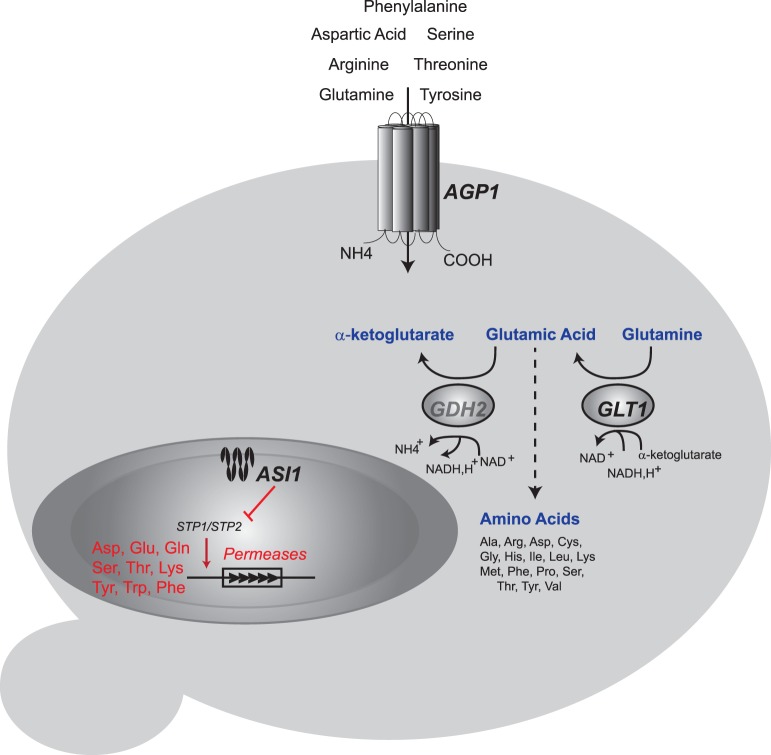
Overview of Nitrogen metabolism QTLs in the WE x SA cross. Genes highlighted in bold were validated by reciprocal hemizygosity and contribute to genetic variation in nitrogen consumption in fermenter yeasts. *GDH2*, which has a FDR = 13%, is shown in light grey. The black arrow represent amino acids differentially consumed in *AGP1* reciprocal hemizygotes, dashed arrow those amino acids produced from glutamic acid and those amino acids with significantly different consumption levels in *ASI1* reciprocal hemizygotes are highlighted in red. Red arrow indicates the suggested differential allelic regulation on those genes encoding for amino acid permeases through the indirect effect of *ASI1*.

Optimal nitrogen assimilation is a crucial phenotype for an efficient fermentation [13]. As described above and other studies, it is affected by many genes, which can also vary among strains [13], [27]. We have described three alleles that affect the consumption of many amino acids and altogether, influencing the competence of the strains to ferment in low nitrogen conditions (**Figure 5**). However, these alleles only account for a small fraction of the overall trait variance, indicating that other loci are also segregating in the WE × SA cross. The identification of these variants offers concrete evidence of the genetic basis for understanding how nitrogen is differentially assimilated upon the source. Furthermore, the results of this study are applicable to other industrial quantitative trait studies and represent a plausible source of genetic material for the implementation of practical approaches towards more efficient strains for nitrogen consumption.

## Materials and Methods

### Yeast Strains

Haploid parental strains DBVPG6765 (WE, *Mat a*, *ho::HygMX*, *ura3::KanMX*) and Y12 (SA, *Mat alpha ho::HygMX*, *ura3::KanMX*) together with the F1 Hybrid and 96 segregants utilized in this study were previously described [32], [55]. All the strains used in this work were short term maintained on YPDA solid media (2% glucose, 0.5% peptone, 0.5% yeast extract, 2% agar).

### Fermentation and Phenotypic Analysis

Fermentations were carried out in duplicates in synthetic wine must (MS300), prepared according to Rossignol *et al* [56] and explained in detail in Table S4. Briefly, MS300 was supplemented with a final concentration of 300 mgN/L of assimilable nitrogen (YAN) corresponding to 120 mgN/L of ammonium and 180 mgN/L of a mixture of 19 amino acids (612.6 mg/L L-proline, 503.5 mg/L L-glutamine, 503.5 mg/L L-arginine monohydrochloride, 179.3 mg/L L-tryptophan, 145.3 mg/L L-alanine, 120.4 mg/L L-glutamic acid, 78.5 mg/L L-serine, 75.92 mg/L L-threonine, 48.4 mg/L L-leucine, 44.5 mg/L L-aspartic acid, 44.5 mg/L L-valine, 37.9 mg/L L-phenylalanine, 32.7 mg/L L-isoleucine, 50.0 mg/L L-histidine monohydrochloride monohydrate, 31.4 mg/L L-methionine, 18.3 mg/L L-tyrosine, 18.3 mg/L L-glycine, 17.0 mg/L L-lysine monohydrocloride, and 13.1 mg/L L-cysteine). The strains were initially grown under constant agitation in 10 ml of MS300 during 16 hours at 25°C. Next, 1×10^6^ cells/mL were inoculated into 12 ml of MS300 (in 15 mL conical tubes) an incubated at 25°C, with no agitations for 6 days, stage at which most nitrogen consumption differences can be observed [34].

After the 6 days, 12 mL of synthetic grape must (MS300) were centrifuged at 9000×*g* for 10 min and the supernatant was collected. 20 µL of MS300 were injected in a Shimadzu Prominence HPLC equipment (Shimadzu, USA) using a Bio-Rad HPX –87H column according to Nissen et al. [57]. The concentration of each amino acid was measured using the HPLC analysis as previously described [58]. The consumption of each nitrogen source was estimated as the difference between the initial and final amounts of each source before and after fermentation, respectively.

### Statistical Analysis

Principal Component Analysis was performed using R software version 2.15 and the prcomp function [59]. Similarly, other statistical analyses, including analysis of variance (one-way ANOVA using the aov function and a simple phenotype ∼ genotype model), and q-value correction (q-value package), were carried out with R software version 2.15. The percentage of transgressive segregants was calculated as previously described [60].

### Linkage Analysis

Linkage analysis was performed as previously described [32] using the rQTL software [61] and LOD scores calculation by a non-parametric model. The significance of a QTL was determined from permutations. For each trait and cross, we permuted the phenotype values within tetrads 1000 times, recording the maximum LOD score each time. We called a QTL significant if its LOD score was greater than the 0.05 tail of the 1000 permuted LOD scores. The percentage of phenotypic variance explained for a QTL was calculated using rQTL [60].

### Reciprocal Hemyzigosity Assay

The genomic intervals of each QTL were examined in the Saccharomyces genome database (SGD), the Gene Ontology (GO) term database [62], the SGRP database [38] and the YRC database [51]. The sequence of the candidates genes were downloaded from the SGRP BLAST server of the University of Toronto (http://www.moseslab.csb.utoronto.ca/sgrp/). To validate the presence of a QTL, we performed a reciprocal hemizygosity assay [63]. The gene *URA3* (essential for pyrimidine biosynthesis) previously deleted in the parental strains [55] was used as a selectable marker with some modifications. Briefly, we used haploid versions of the parental strains (WE *MAT a*, *ho::HygMX*, *ura3::KanMX* and SA *MAT alpha*, *ho::NatMX*,*ura3::KanMX*) to delete each target gene and construct all possible combinations of single deletions. Next, mutated parental strains were crossed to generate the reciprocal hemizygote strains and selected in double drugs plates (50 mg/mL Hygrormycin B and 100 mg/mL Nourseothricin). The diploid hybrid strains were confirmed by *MAT* locus PCR [64] and the deletions of the target genes were confirmed by PCR using the primers pairs A1/S8 o A4/S5 [26]. Primers are listed in **Table S5**. Complete alignment was performed using ClustalX2 (Complete alignment option) and the phylogenetic tree was built using the maximum likelihood algorithm and Tamura-Nei model to estimate genetic distance in PhyML 3.0 software [65].

## Supporting Information

Figure S1 **Amino acid and ammonium consumption distribution across segregants for each trait.**
(PDF)

Figure S2 **Consumption levels in segregants for preferred sources in WE and SA parental strains.** **A.** Glutamine consumption levels in segregants carrying either WE or SA alleles for QTL9. **B.** Ammonium consumption levels in segregants carrying either WE or SA alleles for QTL6.
(PDF)

Figure S3 **Maximum Likelihood tree on the SGRP strains based on** ***GLT1*** **,** ***AGP1*** **and** ***ASI1*** **concatenated sequences.**
(PDF)

Table S1 **Consumption levels (mg/L) for each nitrogen source in the 96 segregants from the WE** × **SA cross.**
(XLSX)

Table S2 **QTLs detected.**
(XLSX)

Table S3 **Consumption levels (mg/L) in reciprocal hemizygotes for** ***GDH2*** **,** ***GLT1*** **,** ***AGP1*** **,** ***GAT2, AGP3*** **and** ***ASI1*** **.** /WE denotes hemizygotes carrying the WE allele, while SA/Δ denotes hemizygotes carrying the SA allele.
(XLSX)

Table S4 **Synthetic must MS300 composition.** Nitrogen, salts, vitamins, anaerobiosis factors and sugar contained in the MS300 medium.
(XLSX)

Table S5 **Primers used in this study. A.** Primers for reciprocal hemizygosity. **B.** Primers for confirmation of the hemizygotes strains.
(XLSX)
