# Supplementary figures and images for: Mapping Genetic Variants Underlying Differences in the Central Nitrogen Metabolism in Fermenter Yeasts
Source: PLoS One. 2014 Jan 21;9(1):e86533. doi: 10.1371/journal.pone.0086533 (PMC3897725; doi:10.1371/journal.pone.0086533)

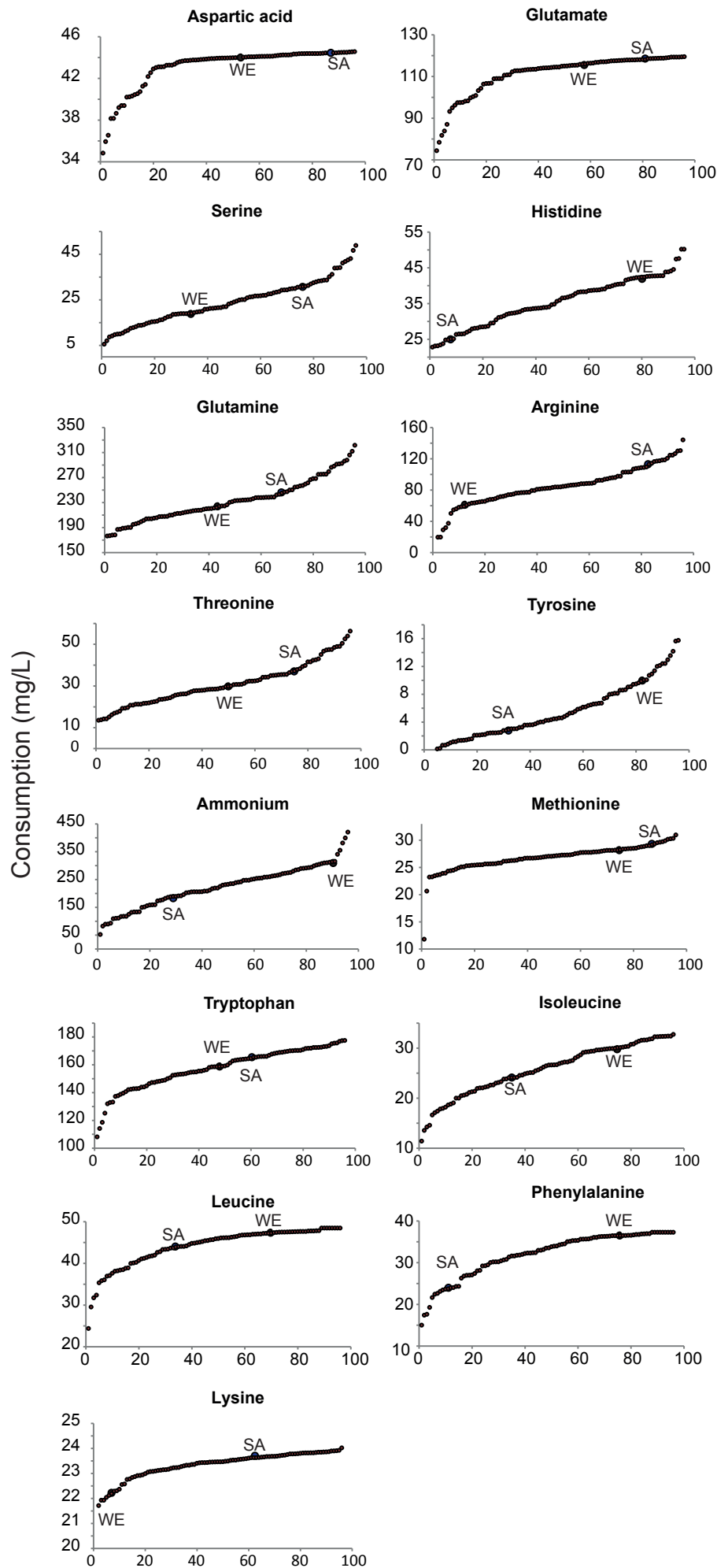

**Figure S1**

Supplement: Figure S1 — Amino acid and ammonium consumption distribution across segregants for each trait. (PDF) [file pone.0086533.s001.pdf]

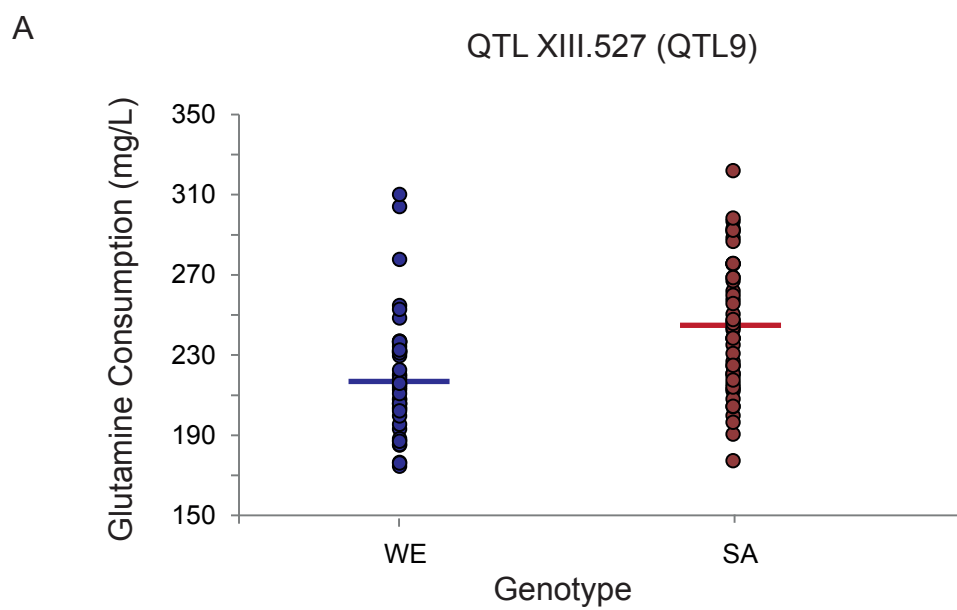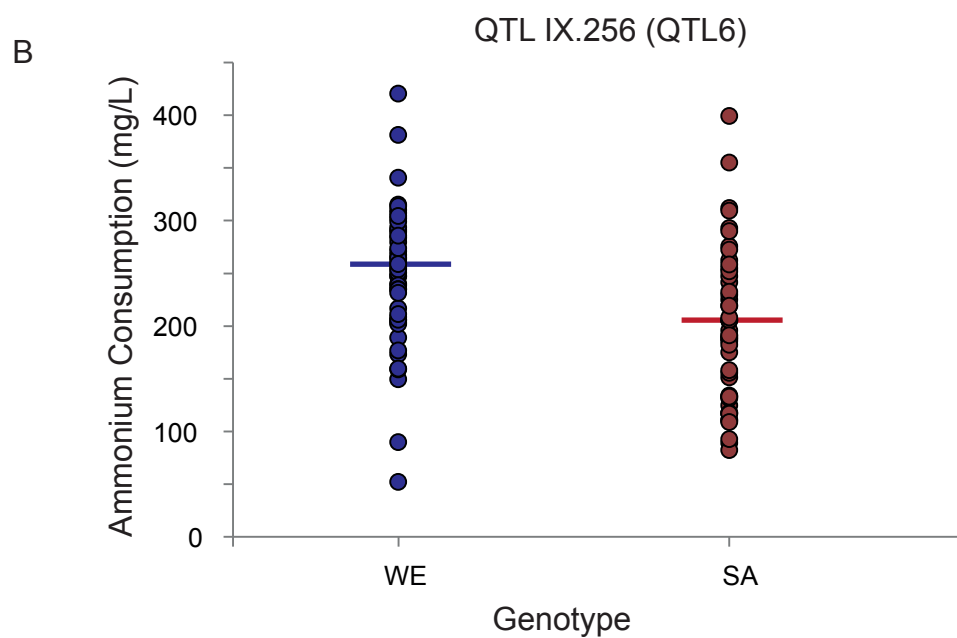

Figure S2

Supplement: Figure S2 — Consumption levels in segregants for preferred sources in WE and SA parental strains. A. Glutamine consumption levels in segregants carrying either WE or SA alleles for QTL9. B. Ammonium consumption levels in segregants carrying either WE or SA alleles for QTL6. (PDF) [file pone.0086533.s002.pdf]

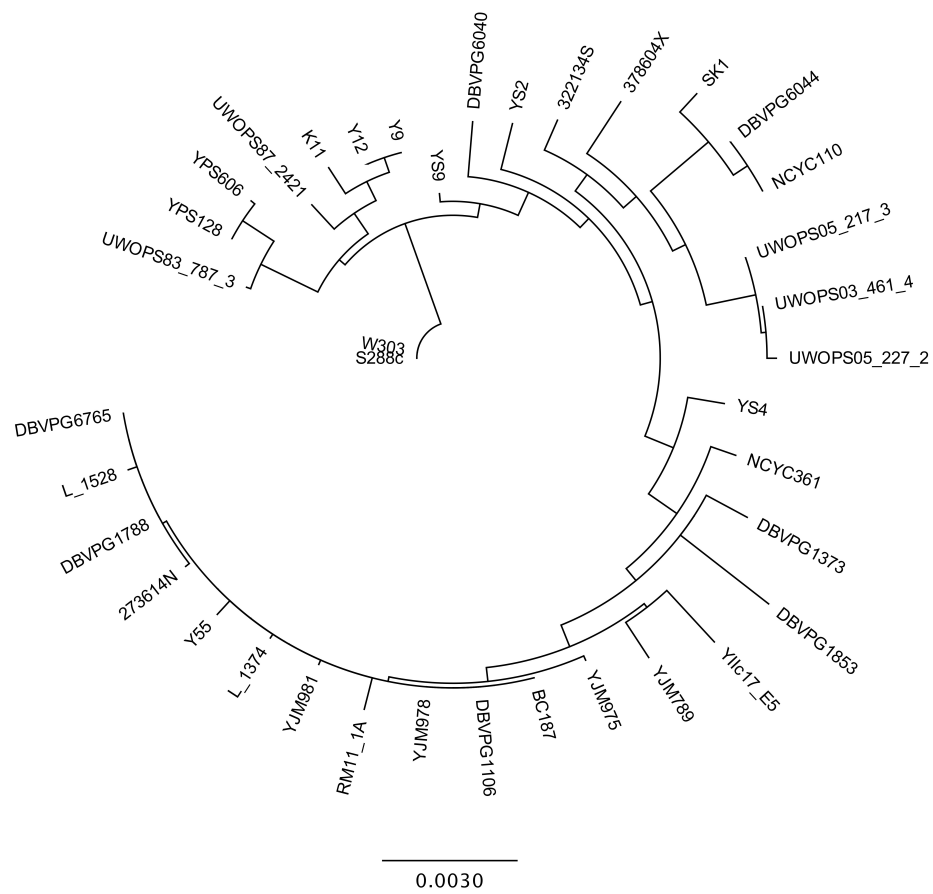

Figure S3

Supplement: Figure S3 — Maximum Likelihood tree on the SGRP strains based on GLT1 , AGP1 and ASI1 concatenated sequences. (PDF) [file pone.0086533.s003.pdf]
